# Pharmacokinetic and pharmacodynamic evaluation of the atypical tetracyclines chelocardin and amidochelocardin in murine infection models

**DOI:** 10.1128/spectrum.01289-23

**Published:** 2023-12-04

**Authors:** Katharina Rox, Rolf Jansen, Tadeja Lukežič, Marina Greweling-Pils, Jennifer Herrmann, Marcus Miethke, Stephan Hüttel, Fabienne Hennessen, Antoine Abou Fayad, Cornelia Holzhausen, Carina Vingsbo Lundberg, Joanne Teague, Enge Sudarman, Lisa Bülter, Thomas Hesterkamp, Marc Stadler, Mark Brönstrup, Rolf Müller

**Affiliations:** 1 Department of Chemical Biology, Helmholtz Centre for Infection Research (HZI), Braunschweig, Germany; 2 German Centre for Infection Research (DZIF), Partner Site Braunschweig-Hannover, Braunschweig, Germany; 3 Department of Microbial Drugs, Helmholtz Centre for Infection Research (HZI), Braunschweig, Germany; 4 Department of Microbial Natural Products, Helmholtz Institute for Pharmaceutical Research Saarland (HIPS), Helmholtz Centre for Infection Research (HZI) and Department of Pharmacy, Saarland University Campus, Saarbrücken, Germany; 5 Mouse Pathology, Helmholtz Centre for Infection Research (HZI), Braunschweig, Germany; 6 Statens Serum Institut, Copenhagen, Denmark; 7 Evotec Ltd, Manchester, United Kingdom; 8 Translational Product Development Office, German Centre for Infection Research (DZIF), Partner Site Braunschweig-Hannover, Braunschweig, Germany; Innovations Therapeutiques et Resistances, Toulouse, France

**Keywords:** tetracycline, urinary tract infection, *Klebsiella pneumoniae*, *Escherichia coli*, PK/PD, chelocardin, amidochelocardin, neutropenic thigh infection

## Abstract

The quest for novel anti-infectives against drug-resistant pathogens of the so-called ESKAPE panel is accompanied by intensive research aiming to find treatment options for the future. In this study, we evaluated the pharmacokinetics and pharmacodynamics of the two atypical tetracyclines: chelocardin (CHD) and amidochelocardin (CDCHD). Although CHD was in phase II clinical trials in the 1970s against urinary tract infections (UTI), CDCHD is a novel derivative obtained by biosynthetic engineering. A pharmacokinetic evaluation in uninfected, non-neutropenic CD-1 outbred mice using intravenous, peroral, and subcutaneous routes showed that CHD had higher plasma exposure than CDCHD but underwent an epimerization that was not observed for CDCHD. CDCHD showed persistently high exposure levels in urine lasting for more than 24 hours, whereas CHD urine concentrations decreased faster over time. Pharmacodynamic characterization in the neutropenic thigh infection model with *K. pneumoniae* and *E. coli* as challenge pathogens in CD-1 outbred mice proved that CHD was more effective in reducing bacterial burden in the thigh, in particular against *E. coli*, whereas CDCHD effectively reduced bacterial burden in kidneys affected by hematogenous seeding from the primary inoculation site, that is, thigh. Assessment of both atypical tetracyclines in an ascending UTI model with bladder as the primary inoculation site against gentamicin as positive control revealed high effectiveness of CDCHD. In summary, CDCHD warrants further preclinical exploration for the indication of UTI.

**IMPORTANCE:**

There is a strong need to find novel treatment options against urinary tract infections associated with antimicrobial resistance. This study evaluates two atypical tetracyclines, namely chelocardin (CHD) and amidochelocardin (CDCHD), with respect to their pharmacokinetics and pharmacodynamics. We show CHD and CDCHD are cleared at high concentrations in mouse urine. Especially, CDCHD is highly effective in an ascending urinary tract infection model, suggesting further preclinical evaluation.

## INTRODUCTION

Antimicrobial resistance (AMR) and the continuous decline in the development of novel antibiotics is a growing threat to society. It is estimated that deaths due to AMR, determined as 1.3 million for 2019, may increase up to 10 million per year in 2050 (1, 2). Thus, novel treatment options against multidrug-resistant bacteria are urgently needed, in particular against the so-called ESKAPE pathogens (*Enterococcus faecium*, *Staphylococcus aureus*, *Klebsiella pneumoniae, Acinetobacter baumannii*, *Pseudomonas aeruginosa,* and *Enterobacter* species) (3, 4).

In antibiotic drug discovery and development, natural products have always been a rich source for novel compound classes exhibiting new mechanisms of action (5 – 7). Despite increasing efforts of isolating novel compounds from plants, fungi, and bacteria such as *Streptomyces* spp., the number of compounds exhibiting novel structures and innovative targets decreases (8, 9). Therefore, one strategy builds on the reassessment of already known but neglected natural product scaffolds. Their pharmacological and microbiological properties could then further be optimized by semi-synthesis and genetic engineering (10).

Amidochelocardin (2-carboxamido-2-deacetyl-chelocardin, CDCHD) is one such example where directed biosynthetic engineering was applied to generate a novel improved derivative of the natural product, that is, chelocardin (CHD) (11 – 13). CHD itself has already been described in the early 1970s (14). It is an atypical tetracycline, which exhibits broad-spectrum antibacterial activity, but, at the same time, is thought to differ from the tetracyclines with respect to the mode of action (15, 16). CHD was tested in a small phase II clinical study in patients with urinary tract infections and exhibited promising activity but was not developed to the stage of market approval (17). CHD showed certain spectrum gaps that could be overcome by CDCHD, for example, anti-*Pseudomonas* activity *in vitro*. In recent studies, it was additionally shown that CDCHD exhibited resistance-breaking properties with respect to tetracycline resistance, serving as the starting point for further analogs (18) and could, thus, be a treatment option for tetracycline-resistant bacterial infections (19).

In this study, we investigated the pharmacological profile of both CHD and CDCHD in detail. Although CHD has already been under clinical investigation (17), no further data about its pharmacokinetic (PK) and pharmacodynamic (PD) properties are available in the public domain. First, we characterized the PK profile of both compounds to select the best route of administration for subsequent *in vivo* efficacy studies. Next, we determined their potency against *K. pneumoniae* and *E. coli* in a neutropenic thigh infection model. Finally, we deployed an ascending urinary tract infection model with *E. coli* to elucidate their potential as treatment options in this specific indication. The results of this pharmacological characterization highlight the potential of both atypical tetracyclines for further preclinical exploration.

## RESULTS

### Pharmacokinetic characterization of CHD and CDCHD

**Fig 1:**
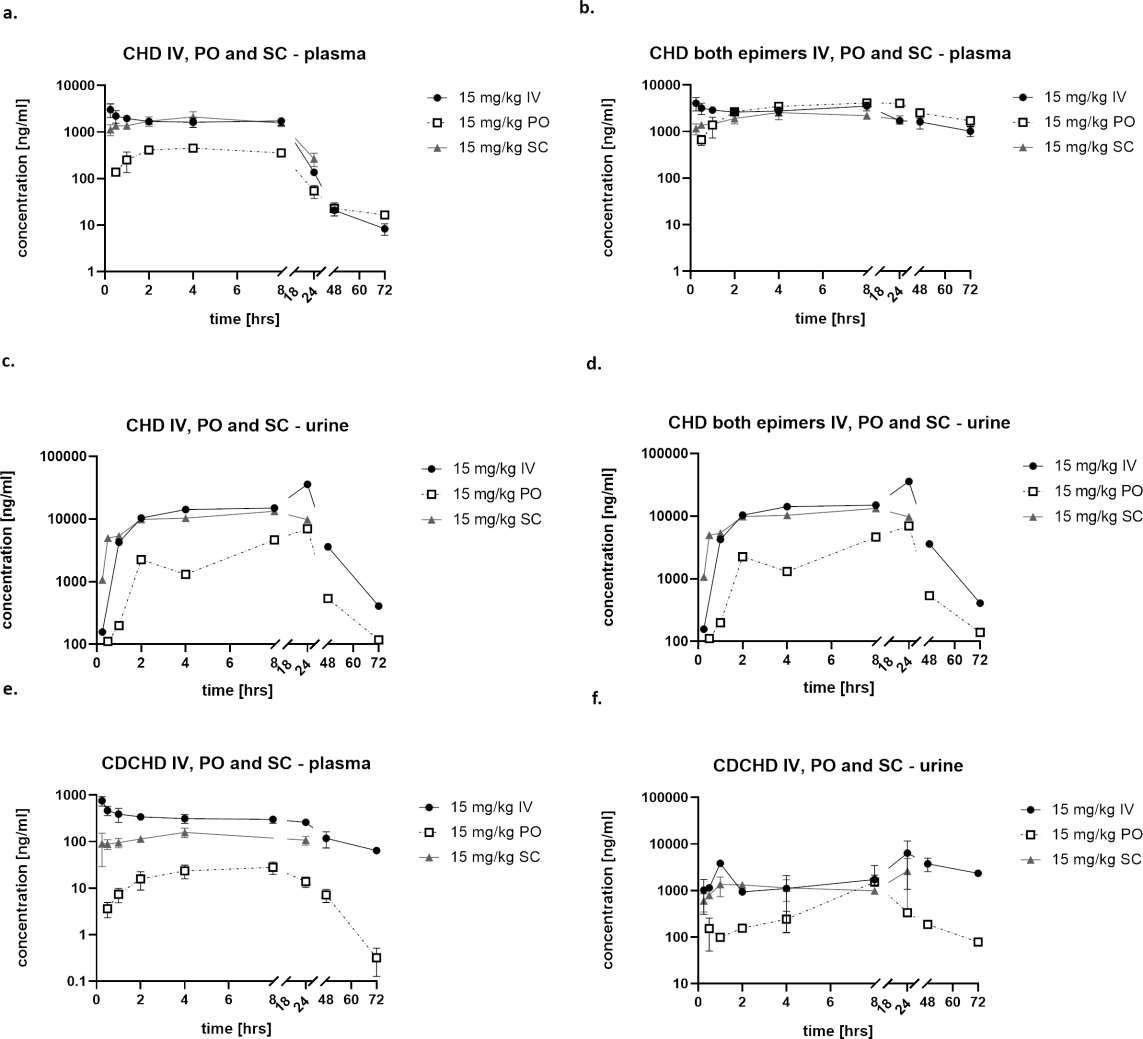
Pharmacokinetic profiles of CHD and CDCHD using different routes of administration. CHD (**a–d**) and CDCHD (**e, f**) were administered at 15 mg/kg IV, PO, and SC, respectively. Plasma concentrations of the active CHD (**a**) and the sum of the active CHD and the inactive epi-CHD (**c**) are displayed. Moreover, urine concentrations of the active (**b**) and the sum of the active CHD and the inactive epi-CHD (**d**) are displayed. Plasma (**e**) and urine (**f**) concentrations of CDCHD are displayed. *N* = 3 mice were used per time point.

We determined the PK profiles of CHD and CDCHD at a dose of 15 mg/kg after intravenous administration and assessed plasma and urine levels at different time points until 72 hours. For tetracyclines, it is known that they are prone to epimerization in position C-4 leading to the formation of less active so-called epi-tetracyclines (20). Importantly, epimerization was also observed for CHD in plasma and to a lower extent in urine. This was not the case for CDCHD (Fig. S1). Epi-CHD was 4- to 16-fold less active than CHD against *E. faecium*, *S. aureus,* and *K. pneumoniae,* whereas both epimers were inactive against *A. baumannii*, *P. aeruginosa,* and *E. aerogenes* (Table S1). For CHD, the plasma and urine levels of the active CHD as well as the sum of active CHD plus epi-CHD were determined. Accordingly, PK parameters were assessed for the active CHD only. CHD showed moderate but sustained plasma levels with a C0 at more than 4 µg/mL. Plasma levels slowly decreased up to the time point t = 72 hours, presumably also due to increased epimer formation over time (Fig. 1a and b). High concentrations of the active CHD were already found 1 hour after administration in urine (Fig. 1c). Despite high epi-CHD concentrations in plasma at 72 hours, epi-CHD was only found at low concentrations in urine at that time point. This could be due to a dynamic equilibrium of CHD and epi-CHD depending on the respective matrix, or to enhanced urinary clearance of epi-CHD (Fig. 1d). Despite a four-fold lower C0 (around 1.3 µg/mL) after IV administration, CDCHD exhibited sustained plasma concentrations at later time points (until 72 hours), 10-fold higher than those observed for the active CHD at time points > 24 hours (Fig. 1E). CDCHD was also found at high concentrations at time points > 24 hours in urine, whereas CHD urine concentrations already declined (Fig. 1f). Because of the epimer formation observed for CHD, the half-life was much lower compared to CDCHD (8.2 vs 24.3 hours, Tables 1 and 2). Moreover, CDCHD exhibited a high volume of distribution, suggesting extensive distribution toward tissues and the intracellular compartment. Finally, CDCHD had around three-fold lower plasma exposure compared to the active CHD (Tables 1 and 2).

**TABLE 1:** PK parameters of CDCHD after 15 mg/kg IV, PO, and SC administration^*a*^

CDCHD	15 mg/kg IV	15 mg/kg PO	15 mg/kg SC
t_1/2_ (h)	24.3 ± 5.2		
C0 (ng/mL)	1,336.4 ± 523.5		
C_max_ (ng/mL)		29.1 ± 8.5	171.7 ± 17.7
T_max_ (h)		7.3 ± 1.6	2.8 ± 2.2
AUC_0-t_ (ng/mL*h)	13,229.0 ± 1,147.2	780.9 ± 187.8	3,109.3 ± 592.0
MRT (h)	33.4 ± 8.0	27.9 ± 7.1	57.3
Vz/F_obs (L/kg)	33.4 ± 6.1	394.5 ± 122.6	119.2
Cl/F_obs (mL/min/kg)	16.0 ± 1.4	294.3 ± 99.2	34.5
F (%)		5.9	23.5

^*a*^ t_1/2_: plasma half-life; C0: concentration at time point t = 0; T_max_: time point at which maximal concentration is reached; C_max_: maximal concentration; AUC_0-t_: area under the curve from time point 0 until t; MRT: mean residence time; Vz/F_obs: fractionated observed volume of distribution; Cl/F_obs: observed fractionated plasma clearance; IV: intravenously; PO: perorally; SC: subcutaneously.

**TABLE 2:** PK parameters of CHD after 15 mg/kg IV, PO, and SC administration^*a*^

CHD	15 mg/kg IV	15 mg/kg PO	15 mg/kg SC
t_1/2_ (h)	8.2 ± 0.3		
C_0_ (ng/mL)	4,234.3 ± 1,737.1		
C_max_ (ng/mL)		460.5 ± 58.6	2,109.3 ± 601.9
T_max_ (h)		3.3 ± 1.2	4.0 ± 0.0
AUC (ng/mL*h)	31,550.8 ± 2,722.6	7,636.3 ± 840.7	28,457.0 ± 4,506.2
MRT (h)	8.5 ± 0.2	19.1 ± 4.1	9.7 ± 1.8
Vz/F_obs (L/kg)	5.6 ± 0.5	55.5 ± 14.5	4.8 ± 1.5
Cl/F_obs (mL/min/kg)	7.9 ± 0.6	31.0 ± 4.3	8.1 ± 1.1
F (%)		24.2	90.2

^*a*^ t_1/2_: plasma half-life; C0: concentration at time point t = 0; T_max_: time point at which maximal concentration is reached; C_max_: maximal concentration; AUC_0-t_: area under the curve from time point 0 until t; MRT: mean residence time; Vz/F_obs: fractionated observed volume of distribution; Cl/F_obs: observed fractionated plasma clearance; IV: intravenously; PO: perorally; SC: subcutaneously.

**Fig 2:**
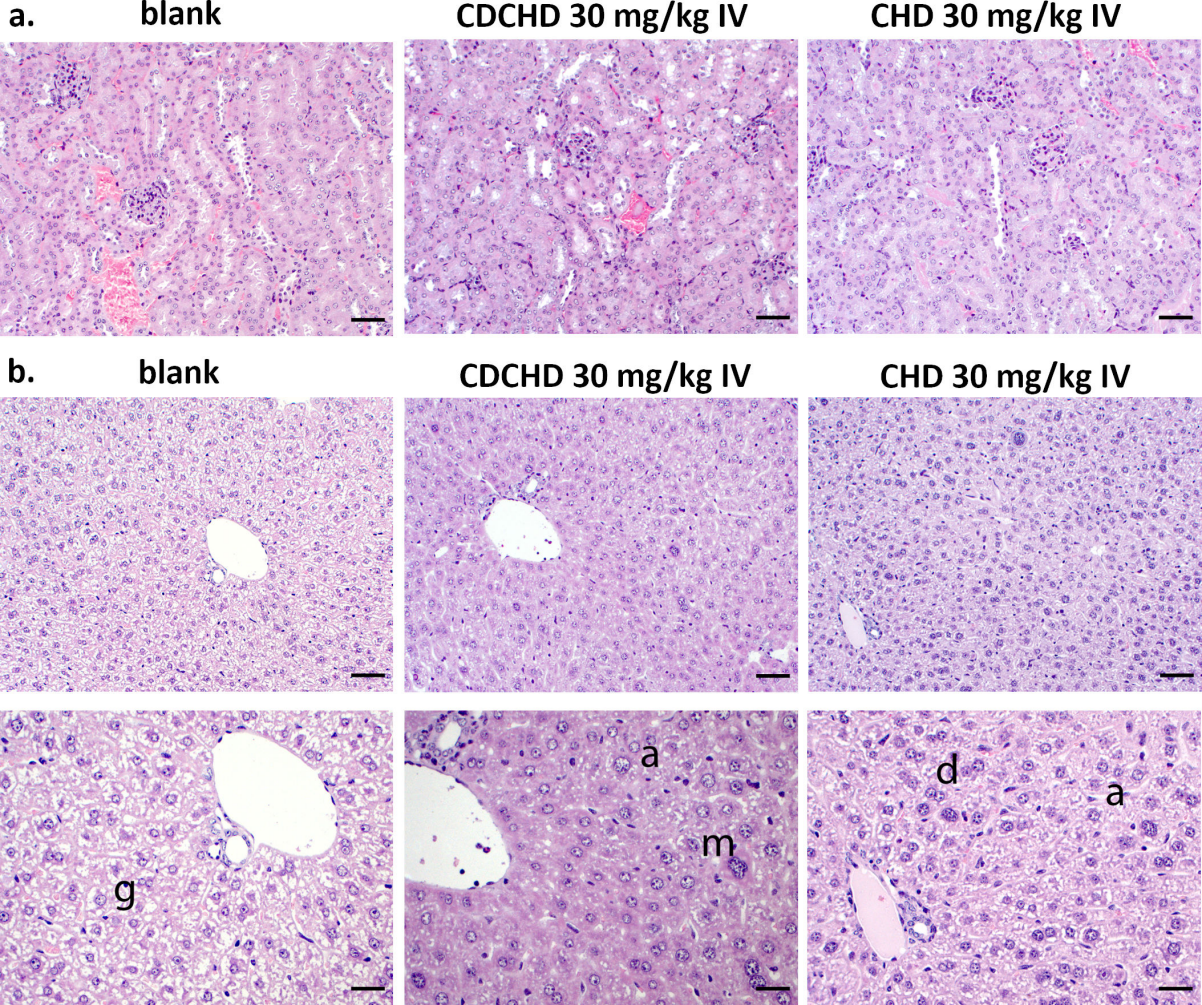
Histopathological analysis of kidneys and livers after high-dose administration of CHD and CDCHD. CHD and CDCHD were administered at 30 mg/kg IV. Twenty-four hours after administration kidneys (**a**) and livers (**b**) of untreated animals (blank) as well as animals treated with CHD and CDCHD were evaluated. Scale bar: 50 µm for (**a**) and upper panels of (**b**); scale bar: 25 µm for lower panels of (**b**). g: normal amount of glycogen; a: anisokaryosis (inequality in the size of the nuclei of cells); m: multi-nucleated cell; d: double-nucleated cells.

The sustained high urine concentrations observed for CDCHD at 15 mg/kg IV raised the concern that accumulation might occur. We therefore performed histopathological analysis of livers and kidneys. A dose of 30 mg/kg of CHD and CDCHD was administered intravenously, and livers and kidneys were examined 24 hours after treatment to investigate signs of acute toxicity. For CHD, plasma levels were in a similar range as for the 15 mg/kg IV dose. However, increased urine levels of CHD were found. By contrast, urine levels for CDCHD at 30 mg/kg IV were in a similar range despite increased plasma levels (by a factor of more than two). This suggested that for CHD, a ceiling effect was already observed at 30 mg/kg IV (Fig. S2). Kidneys of the CHD- and CDCHD-treated groups showed the yellow tissue discoloration of the drugs reported for other tetracyclines before. However, a histopathological analysis did not detect tissue alterations, suggesting no immediate overt toxicity (Fig. 2a). Examination of liver samples revealed sporadic extramedullary erythropoiesis and thrombopoiesis for CHD and CDCHD at 30 mg/kg IV, indicating increased metabolism in the liver. In addition, a moderate reduction of glycogen, moderate anisokaryosis, and a moderate amount of double-nucleated cells were detected in the CDCHD group (Fig. 2b). These alterations were considered non-adverse so that no immediate toxicity red flags were observed in the high-dose groups of CHD and CDCHD.

Next, we embarked on finding a suitable administration route for efficacy testing *in vivo*. First, we assessed the peroral route. The administration of 15 mg/kg PO of CDCHD resulted in a C_max_ of only 92.17 ng/mL with a T_max_ of 7.3 hours post-administration. Exposure after PO administration was low with a bioavailability of only 6% (Fig. 1e; Table 1). By contrast, CHD showed a more than 15 times higher C_max_ than CDCHD after PO administration with an earlier T_max_ at 3.3 hours (Fig. 1a; Table 2). The exposure to CHD after PO administration was also higher compared to the one of CDCHD, resulting in an overall bioavailability of 24%. Monitoring epimerization of CHD after PO administration showed that much higher plasma levels were obtained for the sum of CHD and epi-CHD, close to the levels reached upon IV administration suggesting an additional pH-dependent epimerization in the gut as a result of the administration route (Fig. 1b). As peroral bioavailability was low for CDCHD and as it was planned to test both compounds head-to-head in efficacy models using the same administration route, additional PK studies were performed at 15 mg/kg SC. Again, CHD showed around 10-fold higher plasma levels compared to CDCHD, with concentrations slowly decreasing until 24 hours (Fig. 1a). Furthermore, CHD showed much higher levels in urine compared to CDCHD, which might be a result of the overall higher exposure of CHD in plasma, which also manifested in a 3 times higher AUC (Tables 1 and 2). CDCHD plasma concentrations peaked at around 3 hours post-administration and reached a C_max_ of 171 ng/mL, whereas CHD plasma concentrations showed a T_max_ at 4 hours post-administration with a C_max_ of around 2.1 µg/mL. Again, CHD showed a much higher bioavailability after SC administration compared to CDCHD (90% vs 24%). In summary, PK studies revealed that CHD showed more favorable PK properties compared to CDCHD in terms of higher exposure, higher bioavailability as well as slow but measurable clearance. In addition, the SC administration route was found to be the best route for further efficacy trials, as it allows frequent administration and has acceptable bioavailability for both compounds.

### Pharmacodynamic evaluation in the neutropenic thigh infection model with *K. pneumoniae* ATCC 43816

Next, we embarked on assessing the efficacy of CDCHD and CHD in a neutropenic thigh infection model with *K. pneumoniae* ATCC 43816, a strain exhibiting minimal inhibitory concentrations (MICs) of 5 µg/mL for CHD and of 1.25 µg/mL for CDCHD (Table S2). Initially, we used two different dose regimens for CDCHD and CHD, levofloxacin as positive control and a vehicle control group. The rationale for the dosing regimens used in these studies was based on high concentrations in plasma and/or urine above the respective MIC. We determined bacterial burden 24 hours after infection in the primary organ thigh but also in the kidney inoculated as a result of secondary seeding, and in blood. A significant reduction of bacterial burden in the thigh was observed for the levofloxacin group (Fig. S3a) but not for the CHD and CDCHD groups. The same was found in blood (Fig. S3b). However, a reduction of bacterial burden was observed in the kidney for the CDCHD 10 mg/kg SC BID group compared to vehicle control. This reduction was similar to the one observed for levofloxacin, although not statistically significant (Fig. S3c).

In the next step, we investigated whether higher doses of CHD might lead to a significant reduction in bacterial burden in any of the target tissues. Therefore, three different dosing regimens for CHD were deployed, ranging from one to four times daily with a similar total dose. As the SC route of administration for CHD and CDCHD resulted in a delayed C_max_ and T_max_ (Fig. 1), we also changed the start of therapy toward 1 hour post-infection to allow that high concentration was reached 2 hours post-infection. Again, no effect was observed in the thigh for any of the three higher dose levels tested for CHD (Fig. S4a). In blood, the bacterial burden was reduced for the 50 mg/kg SC QD group of CHD, which was in a similar range as the one of levofloxacin. However, statistical significance was missed (Fig. S4b). In the kidney, no statistically significant effect was observed; however, a slight reduction of bacterial burden favoring the 50 mg/kg SC QD group was seen for CHD. Moreover, here levofloxacin did not reduce bacterial burden with statistical significance (Fig. S4c).

We also tested CDCHD at higher doses, deploying the same dosage regimens as for CHD. For the 50 mg/kg SC QD dose, a statistically significant reduction of bacterial burden in the thigh was observed (Fig. S5a). However, the bacterial burden was only reduced by approximately one log_10_ unit. In blood, a reduction of bacterial burden was observed for all three CDCHD groups, although it was only significant for the 15 mg/kg TID group (Fig. S5b). Notably, all CDCHD dosage regimens reduced bacterial burden in the kidney to apparent sterilization (Fig. S5c). In summary, only a single, high dose of CDCHD administered once daily was able to reduce bacterial burden in the thigh in the neutropenic thigh infection model with *K. pneumoniae*, whereas CDCHD was effective in kidney tissue, inoculated by secondary seeding, also at lower doses down to 10 mg/kg SC BID.

### Pharmacodynamic evaluation in the neutropenic thigh infection model with *E. coli* ATCC 25922

**Fig 3:**
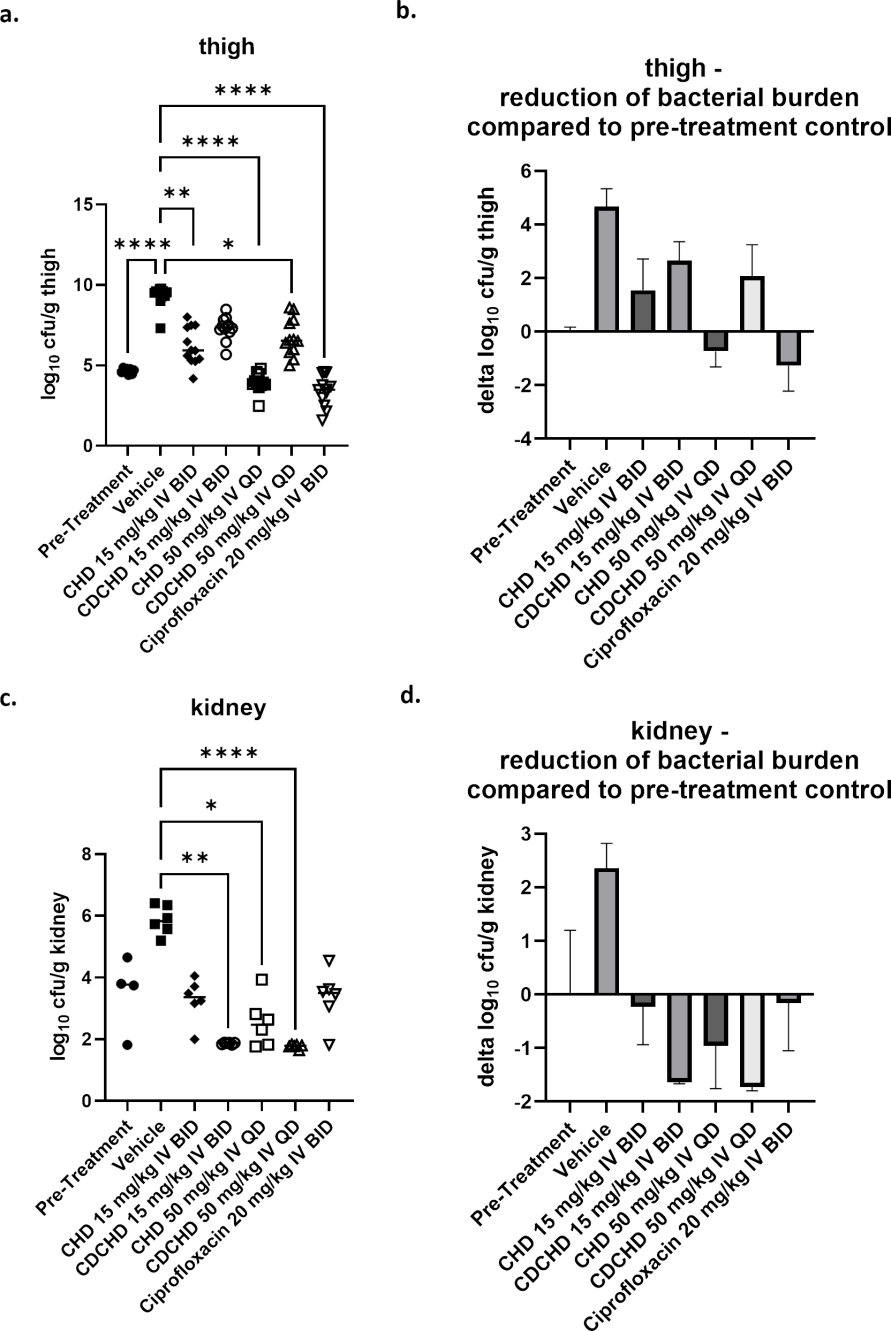
Assessment of low doses of CHD and CDCHD in the neutropenic thigh infection model with *E. coli* ATCC 25922. CHD and CDCHD were tested in the neutropenic thigh infection model with *E. coli* at 15 mg/kg IV BID and 50 mg/kg IV QD, respectively, against ciprofloxacin 20 mg/kg IV BID. Bacterial loads were determined in the thigh (**a, b**) and kidney (**c, d**). Bacterial loads were expressed as log_10_ cfu/g in the thigh and kidney (**a, c**) or as log_10_ reduction compared to the pre-treatment control. Per group *n* = 6 animals were used (12 thighs in total per group), except for the pre-treatment group (*n* = 4). *P < 0.05, **P < 0.01, ***P < 0.001, ****P < 0.0001.

Kidneys are mainly affected by bacteria as a complication of ascending urinary tract infections (21, 22). The most prominent pathogen to cause such infections is *E. coli* (23 – 26). Before probing CHD and CDCHD in a more demanding urinary tract infection model, we first determined whether CHD and CDCHD were effective against *E. coli* ATCC 25922 in the standard neutropenic thigh infection model. CHD and CDCHD exhibited the same MIC (2 µg/mL) against this particular *E. coli* challenge strain (Table S2) (19). Similar to the model with *K. pneumoniae*, the bacterial burden was also determined in kidneys affected by secondary seeding from the thigh. In contrast to the previous assessment, we only used the intravenous route to ensure high initial peak concentrations in plasma and to avoid differences in plasma levels due to distinct bioavailabilities. Moreover, we administered the first dose already 1 hour post-administration to ensure that high plasma levels above the MIC for both compounds were reached shortly after infection. The aim was to enable a head-to-head comparison because both compounds had the same MICs. In addition, we used two different dosage groups and administration schemes for the intravenous route (15 mg/kg BID and 50 mg/kg QD) for CHD and CDCHD. Both compounds exhibited a significant effect when administered intravenously. This was also observed for the low-dose group of CHD administered IV (Fig. 3a). Although both high-dose IV groups of CHD and CDCHD were effective in the thigh, only CHD resulted in a reduction of bacterial burden to levels below stasis, indicating a bactericidal mechanism of action (more pronounced reduction compared to pre-treatment group). However, the positive control ciprofloxacin exerted a stronger effect (Fig. 3b). In kidney, both IV groups of CDCHD (high and low dose) resulted in a pronounced reduction of bacterial burden (Fig. 3c). CHD administered at 15 mg/kg IV BID gave a moderate reduction in kidney, whereas 50 mg/kg IV QD had a significant effect (Fig. 3c). The reduction of CHD and CDCHD observed in kidney for both high-dose IV groups and the low-dose IV CDCHD group was greater than the one seen for ciprofloxacin (Fig. 3d). In summary, both chelocardins exhibited more pronounced effects in kidney in the infection model with *E. coli* ATCC 25922 (MIC for CHD and CDCHD ~2 µg/mL) than in the one with *K. pneumoniae* ATCC 43816 (MIC for CHD at 1.25 µg/mL and 5 µg/mL for CDCHD). Thereby, CDCHD was more effective than CHD.

### Pharmacodynamic effects of CHD and CDCHD in an ascending urinary tract infection model with *E. coli* C175-94

**Fig 4:**
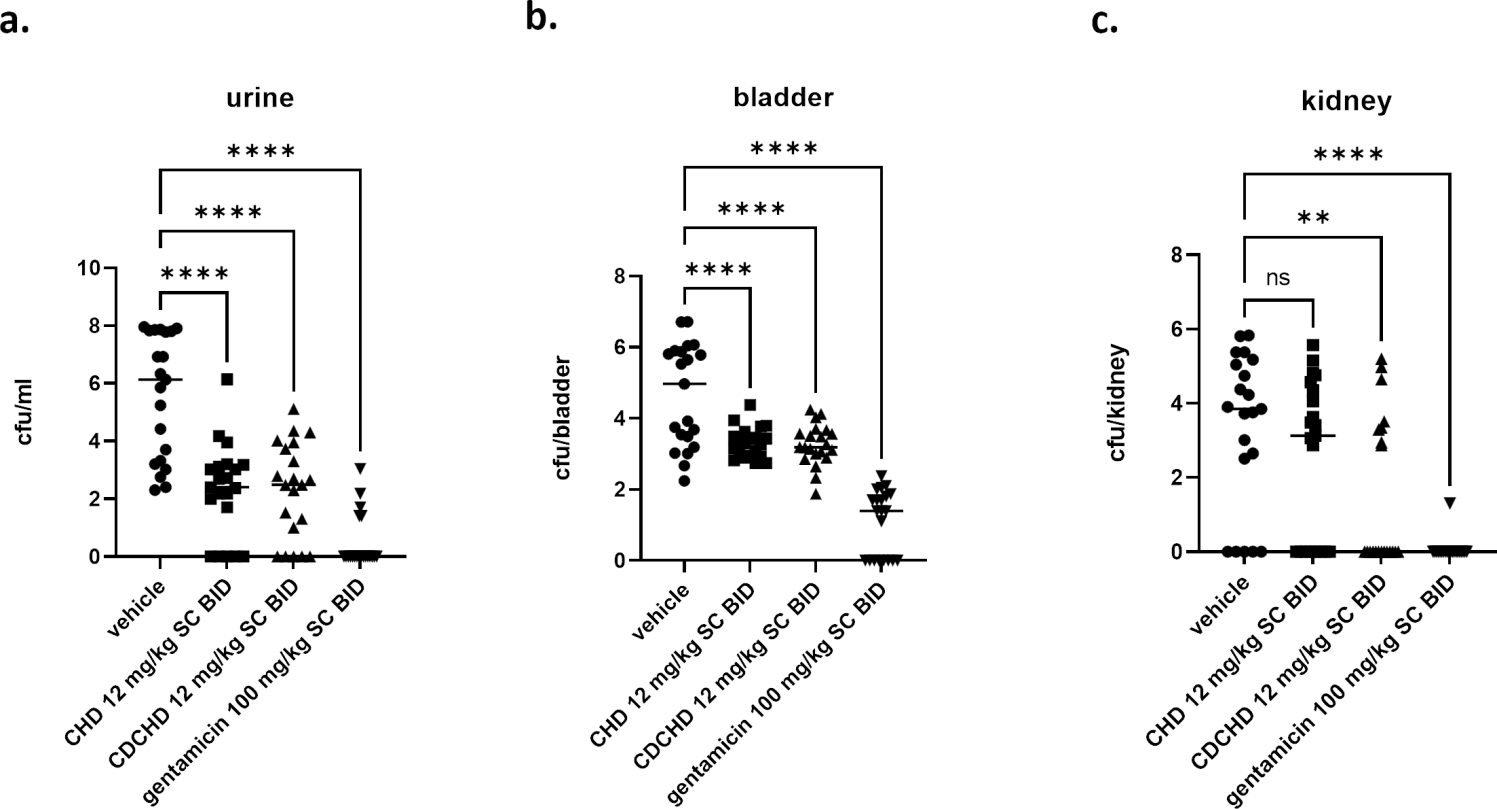
Assessment of CHD and CDCHD in an ascending urinary tract infection model. CHD and CDCHD were tested in an ascending urinary tract infection model with *E. coli* at 10 mg/kg SC BID against gentamicin 100 mg/kg SC BID. Bacterial loads were determined in urine (a), bladder (b), and kidney (c). Bacterial loads were expressed as log_10_ cfu/mL in urine, log_10_ cfu/bladder for bladder, and log_10_ cfu/kidney for kidney. Per group *n* = 21 animals were used. ns : not significant, **P < 0.01, ****P < 0.0001.

**Fig 5:**
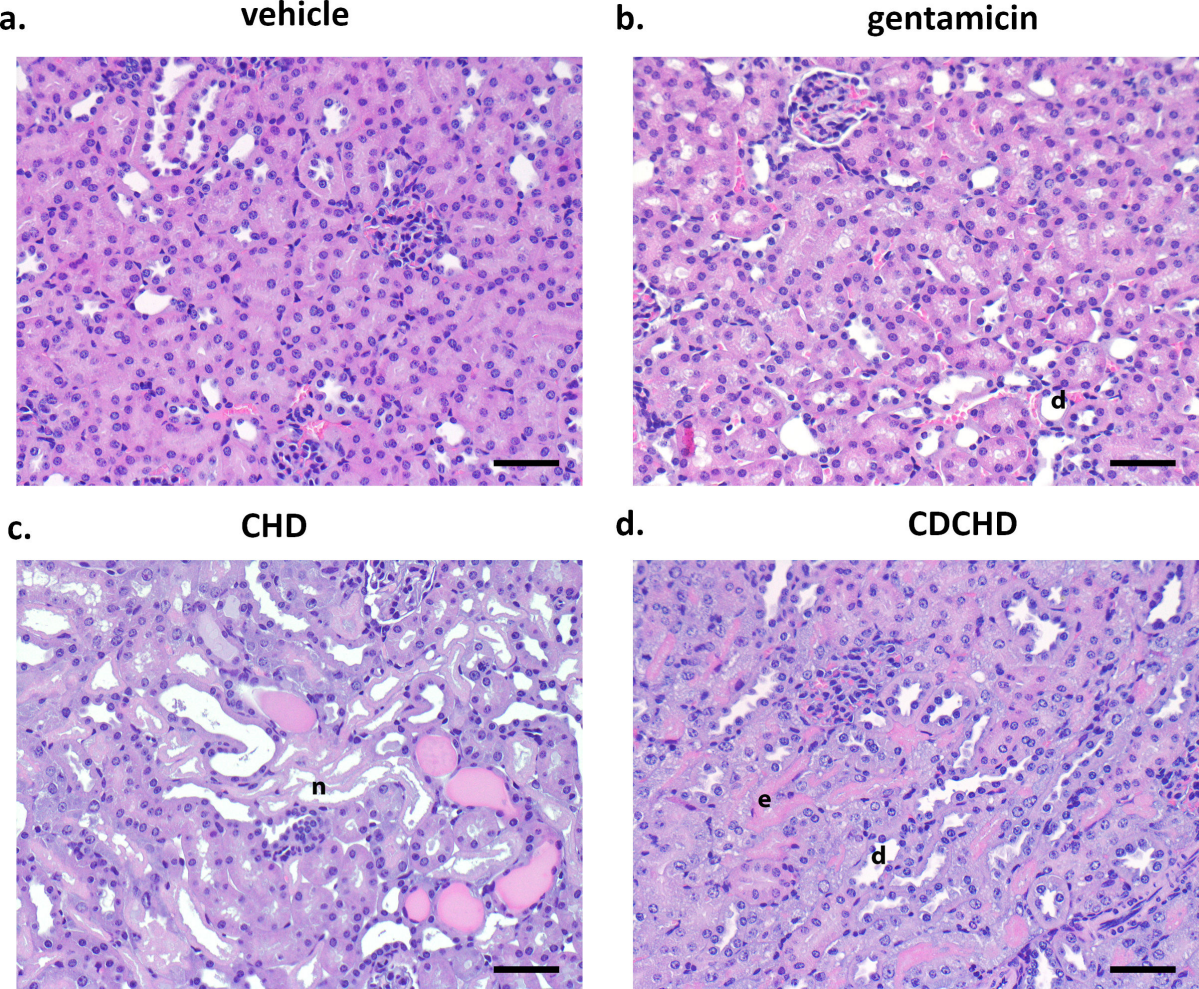
Histopathological analysis of kidneys from CHD, CDCHD, gentamicin, and vehicle-treated groups in an ascending urinary tract infection model. Kidneys of animals from the ascending urinary tract infection model were evaluated at the end of the study. Representative pictures of kidneys of the vehicle-treated (**a**), the gentamicin (**b**), the CHD (**c**), and the CDCHD (**d**) group are displayed. The samples were H&E stained. Scale bars indicate 50 µm. e: eosinophilia, n: tubular necrosis, d: tubular dilation

Based on the encouraging results from the neutropenic thigh infection models with *K. pneumoniae* ATCC 43816 and *E. coli* ATCC 25922 with respect to efficacy in the kidney, we embarked on an ascending urinary tract infection model with the uropathogenic clinical *E. coli* isolate C175-94 (Table S2) (27). Gentamicin was used as a positive control in a very high dose of 100 mg/kg SC BID, and CHD and CDCHD were assessed at 12 mg/kg SC BID. We chose this low dose for CHD and CDCHD as we had already observed bacterial load reductions in the thigh infection models at these doses with good efficiency in the kidney and we intended to differentiate both compounds in a better way in this more complex infection model. Moreover, both compounds did show high levels in urine so we thought that a dose of 12 mg/kg SC BID would be appropriate. Treatment did start one day post-inoculation. Both compounds reduced bacterial burden in urine significantly (Fig. 4a), albeit not as strongly as gentamicin. Using the 95% confidence interval (CI) of the respective means as statistics did confirm the effect observed for the individual data points in urine as the 95% CI for the respective groups did not overlap with the vehicle-treated group (Fig. S6a). In the bladder, both CHD and CDCHD showed a significant reduction compared to vehicle control. The same was observed for gentamicin (Fig. 4b). Moreover, a significant reduction was also observed when applying 95% CI of the mean as descriptive statistics (Fig. S6b). Finally, the bacterial burden in kidneys was determined. Here, gentamicin and CDCHD showed a strong and significant reduction, whereas CHD resulted in a slight, non-significant effect (Fig. 4c). Whereas 23.8% of control animals did have sterile kidneys, this percentage was only slightly higher for CHD (38.1%). CDCHD and gentamicin treatment did result in 61.9% and 95.2% of animals with sterile kidneys, respectively. Similarly, the descriptive statistics with the 95% CI of the means of the vehicle- and the CHD-treated group did show that they were overlapping. Thus, this demonstrated that they were not significantly different. For CDCHD, the 95% CI of the mean did not overlap the one from the vehicle-treated group and was statistically significant. The 95% CI of the mean for the gentamicin-treated group in the kidney was significantly different and much lower compared to the one of the vehicle-treated group (Fig. S6c). CHD and CDCHD exhibited nearly similar terminal plasma concentrations (Fig. S7a). In urine, CHD had slightly higher terminal levels compared to CDCHD (Fig. S7b). Next, we focused on the effects of the compounds in the kidneys and bladder during infection using histopathological analysis. In kidneys, a mild tubulus dilation (with up to 20% of tubuli affected) and a mild to moderate accumulation of inflammatory cells were seen in the renal pelvis in the vehicle-treated group. By contrast, only a mild tubulus dilation without inflammatory cells in the renal pelvis was observed in the gentamicin group. In the CDCHD group, a moderate tubulus dilation (resulting in moderate or 20%–40% of tubuli affected) and protein casts were detected, whereas moderate tubulus degeneration with vacuolated cytoplasm, accumulation of hyaline droplets, protein casts, and a mild to moderate accumulation of inflammatory cells was seen in the renal pelvis in the CHD group (Table 3; Fig. 5). In bladder, mild, focal, submucosal inflammatory aggregates were observed in the CHD and the gentamicin groups, whereas the CDCHD did not show any significant alterations (Table 3). In conclusion, CDCHD and CHD were both effective in the ascending urinary tract infection model. Yet, CDCHD had stronger effects compared to CHD, especially in the kidney. Moreover, in both CHD and CDCHD, a stronger effect on tubulus epithelium was seen compared to gentamicin. As this strong effect was not seen in CHD and CDCHD treatment only (Fig. 2), further studies would be needed to evaluate this effect observed under infection conditions.

**TABLE 3:** Histopathological scoring of kidneys in the ascending urinary tract infection model

Group	No.	Glomeruli	Tubuli	Papilla	Pelvis	Bladder
**CHD**	1 2 3	0 0 0	3 3 1	2 2 0	0 1 2	- 1 1
**CDCHD**	1 2 3	0 0 0	2 2 1	0 0 1	0 1 0	0 0 0
**Gentamicin**	1 2 3	0 0 0	1 1 1	0 0 0	0 0 0	1 0 1
**Vehicle**	1 2 3	0 0 0	1 0 1	0 1 0	1 2 0	0 0 2

## DISCUSSION

The indication of UTIs is facing an increasing need for novel treatment options, in particular for oral formulations (22, 26). Beta-lactam antibiotics and fluoroquinolones, *inter alia* first-line treatment options (28), are either confronted with multidrug-resistant (MDR) bacteria (29) or, in the case of fluoroquinolones, bear the additional risk of rare but severe side effects (30, 31). Recently, the siderophore antibiotic cefiderocol was approved for the indication of complicated UTIs, as it proved to be non-inferior to imipenem-cilastatin (32 – 34). Tetracyclines have been used in the past decades, but “classical” tetracyclines, such as doxycycline or minocycline, are also facing resistance (35) so third-generation tetracyclines, such as the glycylcycline tigecycline, the fluorocycline eravacycline, or the aminomethylcycline omadacycline, were recently developed and approved, albeit in different indications (20). Both CHD and CDCHD are atypical tetracyclines exhibiting no cross-resistance with tetracyclines (36) but not modified at the C-9 position as the third-generation tetracyclines (11, 37).

In this study, we first investigated the PK properties of CHD and CDCHD. We found that both drugs showed similar PK behavior as other third-generation tetracyclines, featured by low plasma levels, low plasma clearance, and long half-life (20). High urine concentrations, although renal excretion does not represent the main excretion pathway, have been described particularly for tigecycline (38) and to a lower extent for omadacycline and eravacycline (39, 40). Thus, similar to the third-generation tetracyclines, CHD and CDCHD also exhibited relatively high concentrations in urine. Histopathological analysis of kidneys, which showed a yellow discoloration at 30 mg/kg IV dosages, confirmed no acute toxicity. Doses up to 50 mg/kg IV as used in the neutropenic thigh infection model were well tolerated. The determination of the maximal tolerated dose (MTD) was out of the scope of this study. However, future studies need to reveal if and at which dose toxicity or adverse effects will be observed. Furthermore, future studies need to investigate if yellow discoloration is also observed at higher doses and if it might cause toxicity above a certain threshold concentration. Extensive epimerization has been described for tetracyclines *in vivo* (41), including the most recently approved congener omadacycline (42). This behavior, independent of the route of administration or dose, was also detected for CHD, but not for CDCHD. This favors CDCHD, as it is known that epimers of tetracycline in position C-4 are less active (20), which was also seen for CHD and epi-CHD. CHD exhibited a higher peroral bioavailability compared to CDCHD. Pronounced differences in peroral bioavailabilities have also been observed for third-generation tetracyclines, resulting in the fact that only omadacycline allows for an IV to PO step-down therapy (40). However, in light of the rapid emergence of resistance in several indications and decreasing treatment options (1, 4), even an intravenous drug may meet unmet clinical needs.

In the neutropenic thigh infection model with *K. pneumoniae* ATCC 43816, only a high dose of CDCHD reduced bacterial burden in the thigh by one log_10_ unit but failed to reduce it to a similar range as the positive control levofloxacin. Taking thigh activity as a proxy for parenchymal antibacterial activity, both drugs may thus fall short of effectively protecting renal tissue in a complicated UTI infection setting. At first sight, it is surprising that CHD did not exhibit an effect, despite the fact that SC administration of CHD resulted in higher plasma levels compared to CDCHD. Although CHD and CDCHD had similar MICs (~4 µg/mL) against a selection of different *K. pneumoniae* strains, CDCHD had a lower minimal bactericidal concentration (MBC; 8 vs 16 µg/mL against *K. pneumoniae* ATCC 43816) (19). Moreover, CDCHD exhibited a four-fold lower MIC compared to CHD against the *K. pneumoniae* ATCC 43816 strain used in the neutropenic thigh infection model. CHD and CDCHD reduced bacterial burden in kidneys that were affected by hematogenous seeding. Whereas CHD showed a slightly higher, but non-significant reduction in kidney compared to levofloxacin, CDCHD reduced bacterial burden back to baseline. This pronounced effect on the kidney as seen for CDCHD might be attributed to active renal concentration, also indicated for CDCHD in the kidney upon macroscopic examination and also supported by high urinary concentrations detected by high-performance liquid chromatography-mass spectrometry (HPLC-MS/MS).

In the neutropenic thigh infection model with *E. coli* ATCC 25922, CHD was more effective than CDCHD, as it already reduced bacterial burden in the thigh at 15 mg/kg IV BID. CDCHD only lowered bacterial burden in thigh at doses of 50 mg/kg IV but did not reduce bacterial load back to stasis. By contrast, CHD at 50 mg/kg IV resulted in a nearly 1 log_10_-unit kill compared to the pre-treatment group and a 5 log_10_-unit reduction compared to the vehicle. The higher efficacy seen for CHD might be explained by the more favorable pharmacokinetic properties (higher exposure and higher C0), as MICs against *E. coli* ATCC 25922 were the same (2 µg/mL) (19). However, in the kidney, the results were reverted, and again CDCHD was more effective in reducing bacterial burden than CHD. These findings might be explained by the pharmacokinetic properties of CDCHD which was not prone to epimerization and displayed high concentrations in urine. Because of high local concentrations, CDCHD reduces bacterial burden in the kidney more efficiently than CHD. CHD and CDCHD were not as effective in the neutropenic thigh infection models as the third-generation tetracyclines, tigecycline, eravacyline, or omadacycline because these resulted in efficient bacterial reductions in the thigh at similar doses (43 – 45). However, these studies did not investigate the effects on organs, such as kidneys, affected by secondary seeding. In this study, CDCHD was even more effective than ciprofloxacin in the kidney.

It is known that gentamicin accumulates in the kidneys and, thus, results in prolonged urine concentrations (27). Therefore, it is a well-established control in urinary tract infection models. As the results for the kidney in the neutropenic thigh infection model with *E. coli* indicated that the 50 mg/kg IV dose was similar to the 15 mg/kg IV dose for CDCHD to reduce bacterial burden efficiently, we chose to dose 15 mg/kg IV BID for CDCHD and CHD in the ascending urinary tract infection model. Whereas CHD had shown a difference that 15 mg/kg IV was inferior to the 50 mg/kg IV dose in a reduction of bacterial burden in kidneys in the neutropenic thigh infection model with *E. coli*, we assumed that BID dosing would be sufficient to achieve high levels for CHD in organs as well. Our results in the ascending urinary tract infection model for CDCHD and CHD demonstrated that high levels in urine were achieved terminally suggesting that this also applied to the kidney and bladder. Future studies will investigate the compound levels in the kidney and bladder to ensure that sufficient concentrations are reached to result in a bactericidal effect in parenchymal bacterial infections. Furthermore, terminal plasma and urine levels determined in the CHD and CDCHD groups in the urinary tract infection model were in line with the projections of terminal PK plasma and urine levels (when taking into consideration BID dosing). This suggested that PK in infected animals was not altered compared to the PK behavior observed in non-neutropenic, uninfected mice. In this model, CHD and CDCHD were slightly inferior to gentamicin in bladder and urine but still showed significant reduction. CHD and CDCHD exhibited remarkable effects, in particular in kidneys, where CDCHD showed a strong reduction of bacterial burden and a high percentage of animals with sterile kidneys. The preliminary histopathological analysis on a limited number of samples revealed that no alterations were detected in the bladder upon treatment with CDCHD, whereas alterations were seen in all other groups, including the gentamicin group. This finding needs further validation with a bigger group size to enable a quantitative analysis of effects as the analysis of only three animals per group can just give an indication of possible effects. CDCHD caused partly moderate and CHD partly severe dilation of the tubulus. Moreover, due to the limited group size and the lack of corresponding cfu data, it cannot be ruled out if alternations are a result of toxicity or due to the infection itself. Therefore, the reversibility of this effect as well as the effect itself observed in this study should be proven in a long-term study in an uninfected setting, preferentially at a high dose of 50 mg/kg IV as the initial histopathological analysis presented here had only been performed for doses up to 30 mg/kg IV. We note that third-generation tetracyclines are already approved or under investigation for the treatment of UTIs. Although tigecycline was proven effective, presumably as a result of high urine concentrations, it is currently questionable whether the risk-benefit ratio favors its application in the indication of UTI (46 – 48). By contrast, for eravacycline and omadacycline, the effectiveness in preclinical urinary tract infection models was shown (49, 50). However, in the IGNITE3 clinical trial, eravacycline did not meet its primary endpoint (51). Omadacycline is still under clinical investigation against UTI.

Recent studies have underlined the resistance-breaking properties of CDCHD and suggest that it will remain active in case of resistance mechanisms already affecting tigecycline, eravacycline, or omadacycline (19, 52). In addition, it was shown that CDCHD retained its activity in the presence of artificial urine (19). Furthermore, the ascending urinary tract infection model demonstrated the efficacy of CDCHD at 10-fold lower doses compared to gentamicin. These findings hold some promise for a further preclinical exploration in the indication of UTI. The next steps should include studies to investigate the transport mechanisms behind high kidney and urine concentrations, particularly seen for CDCHD, and to examine potential adverse effects on kidney tissue after repeat-dosing.

In summary, we present a comparison and PK/PD characterization of the two atypical tetracyclines CHD and CDCHD. Whereas CHD had been investigated until phase II clinical trials against UTI, the biosynthetically engineered CDCHD showed more pronounced pharmacodynamic effects than CHD, opening a perspective for further preclinical studies for the treatment of UTI.

## MATERIALS AND METHODS

### Production of CHD and CDCHD

CHD and CDCHD were each produced in a 15 L bioreactor (bbi-biotech xCUBIO, Berlin) equipped with three Rushton impellers (6-blade Rushton turbines diameter 124 mm). The fed-batch process was performed with the recombinant *Amycolatopsis sulphurea* strain C641#2 pAB03oxyDP. The reactor was inoculated with 1 L of a shake flask pre-culture inoculated with cryogenic tubes of the respective working cell bank. Seed cultures were incubated for 120 hours at 30°C, at 220 rpm on a rotary shaker in CHD-V2 medium consisting of 16,5 g/L glucose monohydrate, 15 g/L soy flour, 1 g/L yeast extract (Biolife), 5 g/L NaCl (Roth), 1 g/L, and CaCO_3_ 0,55 g/L. Before sterilization, the medium was adjusted to pH 7.0 with KOH and H_2_SO_4_.

Batch cultivation was carried out in CHD-F2 medium (55 g/L glucose monohydrate, 20 g/L soy flour, 5 g/L yeast extract, 3 g/L CaCO_3_, 0,55 g/L citric acid monohydrate, pH 7.0) with the addition of 50 g/l XAD-16 adsorber resin (Sigma) for 29 days. Cultivation was performed at 30°C, and a controlled DO value of 20% with a fixed aeration of 0.5 vvm and increasing tip speed (100–400 rpm). The pH value remained unregulated until day 6, afterward it was regulated at pH 7.0 until the end of the process with 5% H_2_SO_4_ and 2.5% KOH. The entire process was monitored with a foam detection probe and controlled *via* antifoam (Tegosipon EVONIK) addition. If the glucose concentration dropped beneath 30 g/L, glucose feeding at a constant rate of 36 g/day was initialized. Feeding was performed with a feeding solution containing 550 g/L glucose monohydrate.

After cultivation, XAD-16 was harvested with 210 µm nylon gaze over a pressure suction filter, and the recovered resin was washed with 5 L of deionized water.

### Isolation of CHD and CDCHD

An amount of 921 g wet Amberlite XAD 1180 (previously referred to us as XAD-16) was transferred to a glass column and washed with a gradient of 3 L of water, 4 L of MeOH/water (60:40), 1 L of MeOH/water (70:30), and 1 L of MeOH/water (80:20). These fractions contained the impurities and were discarded. Finally, the chelocardins were eluted with 4 L of MeOH/water 95:5 buffered with 0.5% trifluoroacetic acid (TFA). After evaporation to 750 mL, the methanol/water mixture was extracted with approximately 2.25 L of heptane in three portions. Evaporation of the methanol layer provided 58.27 g of raw product containing ca. 6 g of CDCHD and 3.21 g of CHD, which was dissolved in 400 mL of MeOH.

This solution was mixed with 200 g of silica gel, evaporated to dryness, mixed with toluene, and again evaporated to dryness thoroughly. The silica gel mixture was transferred to a glass column (9.5 cm x 40 cm) filled with 500 mL of silica gel in dichloromethane (DCM). The column was eluted with a gradient of 1.5 L of DCM, 2 L of DCM/ethanol 9:1, 2 L of DCM/ethanol 8:2, 3 L of DCM/ethanol 7:3, and 6 L of DCM/ethanol 7:3 containing 0.2% of TFA. An intermediate product (A) eluted with DCM/ethanol 7:3 contained 1.4 g of CDCHD in 7.75 g residue while another intermediate product (B) eluted with DCM/ethanol 7:3 containing 0.2% of TFA provided 12.13 g of material with 1.9 g of CDCHD and 0.87 g of CHD.

Intermediate product (B) was subjected to RP-flash chromatography (Reveleris X2 instrument, column Reveleris C18, 330 g; solvent A H_2_O + 0.2% TFA, solvent B acetonitrile +0.2% TFA, gradient 15% B for 5 min, to 40% B in 60 min to 100% B in 15 min; flow 20 mL/min for 3 min, then 130 mL/min. The sample was dissolved in 10 mL of DMSO and 10 mL MeOH +0.2% TFA. Fractions were collected according to UV detection at 290 nm and analyzed by RP-HPLC. A main fraction was evaporated to remove the organic solvent and centrifuged to give a residue of 1.3 g containing 1.17 g of CDCHD (of 90% purity).

Intermediate product A was separated similarly and provided fraction (1) [1.12 g with 995 mg CDCHD (89%)], fraction (2) [369 mg with 198 mg CDCHD and 134 mg CHD], and a fraction (3) [521 mg with 384 mg CHD (74%)].

Fraction (3) was purified in four portions by RP-HPLC [column 250 × 50 mm, Gemini C18, 10 µ, 110 Å (Phenomenex) with pre-column; solvent A H_2_O + 0.2% TFA, solvent B MeOH +0.2% TFA, flow 53% B with 40 mL/min for 52 min and with 50 ml/min for 70 min; each portion was dissolved in 2 mL of DMSO and 2 mL of MeOH and 0.2% TFA]. Fractions were collected by UV at 290 nm and analyzed by RP-HPLC. The fraction containing CHD was evaporated at 32°C water bath temperature, dissolved in MeOH +0.2% TFA, and stored at −22°C. The corresponding four main fractions were combined and evaporated to give 238 mg CHD (of 90.6% purity) and stored at −70°C after evaporation from MeOH +0.6% HCl.

### Preparation of CHD and CDCHD formulations

CHD and CDCHD were prepared as described previously for CHD (53). In brief, monosodium citrate dehydrate (10 mg) was dissolved in water (5 mL). CHD or CDCHD (as HCl salt) was added to the solution and stirred to keep the solution dispersed. Then the solution was cooled down to 4–5 °C and neutralized by adding a stoichiometric (approximately 1 equivalent) amount of sodium hydroxide solution (0.1 M). The resulting pH was between 8 and 9. Then the solution was diluted by adding 50 mL of water and then freeze-dried. After the freeze-drying procedure, the vial was flushed with nitrogen.

### Bacterial strains

The following strains were used for *in vivo* studies or MIC testing: *K. pneumoniae* ATCC 43816, *K. pneumoniae* DSM-30104, *E. faecium* DSM-20477, *S. aureus* DSM-346, *P. aeruginosa* DSM-11128, *A. baumannii* DSM-30008, *E. aerogenes* DSM-30053, *E. coli* ATCC 25922, and *E. coli* C175-94 (27).

### Animals

For pharmacokinetic experiments, outbred, male CD-1 mice (Charles River, Germany), 4-week old, were used; for the neutropenic thigh infection model with *K. pneumoniae* outbred, male CD-1 mice (Charles River, Germany), 6-week old, were used; for the neutropenic thigh infection model with *E. coli* outbred, male CD-1 mice (Charles River, United Kingdom), 6-week old, were used; for the ascending urinary tract infection model outbred, female OF-1 mice (Charles River, France), 8-week old, were used. The animal studies were conducted in accordance with the recommendations of the European Community (Directive 2010/63/EU, 1st January 2013). All animal procedures were performed in strict accordance with the German regulations of the Society for Laboratory Animal Science (GV-SOLAS) and the European Health Law of the Federation of Laboratory Animal Science Associations (FELASA). Animals were excluded from further analysis if the sacrifice was necessary according to the humane endpoints established by the ethical board. All PK studies as well as the neutropenic thigh infection model with *K. pneumoniae* were approved by the ethical board of the Niedersächsisches Landesamt für Verbraucherschutz und Lebensmittelsicherheit, Oldenburg, Germany. The neutropenic thigh infection model with *E. coli* was performed under UK Home Office Licenses and with local ethical committee clearance. The experiment in the ascending urinary tract infection model was approved by the Danish Animal Experimentation Inspectorate.

### Determination of the MIC

The assay was conducted as described previously (54) with the following modifications. The MIC was tested against the *K. pneumoniae* strain ATCC 43816 in an MHB (not cation-adjusted) medium. CHD and CDCHD were tested in concentrations ranging from 0.078 to 10 µg/mL. Moreover, the MIC was determined for the following strains: *K. pneumoniae* DSM-30104, *E. faecium* DSM-20477, *S. aureus* DSM-346, *P. aeruginosa* DSM-11128, *A. baumannii* DSM-30008, *E. aerogenes* DSM-30053, *E. coli* ATCC 25922, and *E. coli* C175-94. All strains were grown in MHB medium, except for *E. faecium*, which was grown in TSB. CHD, epi-CDH, and CDCHD were tested in concentrations ranging from 0.125 to 64 µg/mL as described previously (19).

### PK studies

CHD and CDCHD were administered in single PK studies at the doses of 15 mg/kg and 30 mg/kg IV, at 15 mg/kg SC, and 15 mg/kg PO. Up to 25 µL of blood was collected from the lateral tail vein (*n* = 3 per time point) and time points t = 0.5, 1, 2, 4, and 8 hours. For 15 mg/kg IV- and PO-administered compounds, blood was also collected at time points t = 24 and 48 hours. For IV- and SC-administered compounds, blood was also collected at t = 0.25 hours. At t = 24 hours (15 mg/kg SC, 30 mg/kg IV) or 72 hours (15 mg/kg IV and PO), animals were euthanized to collect blood. At every blood collection, time point spontaneous urine was collected as well. Whole blood was collected into Eppendorf tubes coated with 0.5 M EDTA and immediately spun down at 15,870× *g* for 10 min at 4°C. Then, plasma was transferred into a new Eppendorf tube and stored at −80°C until analysis.

### Bioanalysis of pharmacokinetic and pharmacodynamic samples

First, a calibration curve was prepared by spiking different concentrations of CHD and CDCHD into mouse plasma or mouse urine (matrix for mouse PK or PD samples) from CD-1 mice. Caffeine was used as an internal standard. In addition, quality control samples (QCs) were prepared for CHD and CDCHD in plasma and urine. The following extraction procedures were used: 7.5 µL of a plasma or urine sample (calibration samples, QCs, PK or PD samples) was extracted with 37.5 µL of a mixture of methanol/acetonitrile (2:1) containing 12.5 ng/mL of glipizide as an internal standard for 5 min at 2,000 rpm on an Eppendorf MixMate® vortex mixer. Then samples were spun down at 15,870× *g* for 10 min. Supernatants were transferred to standard HPLC glass vials.

Samples were analyzed using an Agilent 1290 HPLC system coupled to an AB Sciex QTrap 6500 mass spectrometer. LC conditions were as follows: column: Agilent Zorbax Eclipse Plus C18, 50 × 2.1 mm, 1.8 µm; temperature: 30°C; injection volume: 5 µL per sample; flow rate: 700 µL/min. Samples were run under the following conditions. Solvents for acidic conditions: A: 100% water +0.1% formic acid; solvent B: 100% acetonitrile +0.1% formic acid. The gradient for CDCHD was as follows: 99% A at 0 min, 99% A until 1 min, 99%–50% A from 1 to 2.4 min, 50%–35% A from 2.4 to 2.7 min, 35–0% A from 2.7 until 4.5 min, 0% A until 6 min, 0-99% from 6 to 6.2 min, 99% A until 8.0 min. The gradient for CHD was as follows: 99%–90% A from 0 to 1 min, 90%–55% A from 1 to 2.3 min, 50%–40% A from 2.3 to 2.5 min, 40%–0% A from 2.5 to 4.5 min, 0% A until 6 min, 0%–99% A from 6 to 6.2 min, 99% A until 8.0 min. Mass transitions are depicted in Table S3. Peaks of PK and PD samples were quantified using the calibration curve. The accuracy of the calibration curve was determined using QCs independently prepared on different days. PK parameters were determined using a non-compartmental analysis with PKSolver (55).

### PD studies

#### Neutropenic thigh infection model with *K. pneumoniae* ATCC 43816 and *E. coli* ATCC 25922

The inoculum for *K. pneumoniae* ATCC 43816 was prepared freshly as described previously to yield 1 × 10^4^ cfu/mL (56). The inoculum of the *E. coli* ATCC 25922 was diluted from a frozen stock to 1.4 × 10^6^ cfu/ml. *N* = 6 animals per group were used for both tested strains. *N* = 4 mice were used in the pre-treatment group in the neutropenic thigh infection model with *E. coli* ATCC 25922. Mice were rendered neutropenic by administration of 150 mg/kg and 100 mg/kg cyclophosphamide intraperitoneally on days −4 and −1, respectively. On the day of infection (day 0), mice received 30 µL of the inoculum of *K. pneumoniae* ATCC 43816 into each lateral thigh under isoflurane anesthesia, corresponding to 6 × 10^2^ cfu/mouse. In the model with *E. coli*, mice received 50 µL of the inoculum of *E. coli* into each lateral thigh under isoflurane anesthesia, corresponding to 1.4 × 10^5^ cfu/mouse. Whilst still under anesthesia, mice were administered a dose of Buprenorphine (0.03 mg/kg) subcutaneously for pain relief. 24 hours after infection, mice were euthanized by slowly introducing CO_2_ and cardiac puncture to remove blood from the heart immediately after death. Kidneys and thighs were aseptically removed. 24 hours after infection, the clinical score of every individual animal was assessed. Clinical scoring comprises the assessment of different parameters such as spontaneous behavior, posture, appearance, and provoked behavior. Each parameter was assessed with a score of 0 to 3 (ascending severity from 0 to 3). The humane endpoint was reached when the clinical score of a single parameter was 3 or if the total clinical score was higher than 8. Whole blood was collected into Eppendorf tubes coated with 0.5 M EDTA. Organs were homogenized in 0.9% NaCl solution. Organs and blood were plated onto agar plates in duplicates in serial dilutions and incubated at 37°C for 24 hours. 25 µL per dilution of the homogenized organ was spotted onto agar plates. In addition, 1 mL of homogenized organ is streaked onto an agar plate in technical duplicates. If no single colony is observed then, this would result in 0 cfu/mL. Thus, the threshold of detection is 0 log_10_ cfu/g. The groups used in the different neutropenic thigh infection models are found in Tables S4 through S7. In brief, levofloxacin with 3.3 mg/kg TID IP was used as a positive control antibiotic for the *K. pneumoniae* model, whereas ciprofloxacin with 20 mg/kg BID IV was used for the *E. coli* model, respectively. The rationale for TID dosing was based on the PK/PD index of *f*AUC/MIC for fluoroquinolones (57 – 59). As indicated in Table S4, CHD and CDCHD were administered at 10 mg/kg SC BID at t = 2 and 10 hours and at 10 mg/kg SC QD at t = 6 hours. Moreover, CHD and CDCHD were administered at 10 mg/kg SC QID (t = 1,3,5,7 hours), 15 mg/kg SC TID (t = 1,4,7 hours), and 50 mg/kg SC QD (t = 1 hour) (Tables S5 and S6). In the *E. coli* neutropenic thigh infection model, CHD and CDCHD, respectively, were administered at 50 mg/kg IV QD (t = 1 hour) and 15 mg/kg IV BID (t = 1 and 6 hours) (Table S7).

#### Ascending urinary tract infection model with *E. coli* C175-94

The model was conducted as described previously (27, 60). In brief, *n* = 21 animals were used per group in the main study, and *n* = 3 animals per group in satellite groups for histopathological analysis. Three days before the start of the study and during the study, mice had free access to 5% glucose as drinking water. Fresh overnight colonies of *E. coli* from a 5% horse blood agar plate were suspended in PBS pH 7.4 to approximately 10^9^ cfu/mL. Approximately 1 hour before inoculation, mice were treated orally with 45 mL Nurofen (20 mg ibuprofen/mL corresponding to approximately 30 mg/kg) as a pain relief. Urine was removed from the bladder by gently pressing the abdomen and mice were anaesthetized with 0.15 mL Zoletil mix SC. Thereafter, the mice were inoculated. A syringe with a catheter, containing the bacterial suspension was inserted *via* the urethra into the bladder and 50 µL of the inoculum was slowly injected into the bladder. Hereafter, the mouse was placed in the cage and kept in a warming cabinet until fully awake (approximately 4 hours). The mice were treated with CHD (12 mg/kg SC BID), CDCHD (12 mg/kg SC BID), gentamicin (100 mg/kg SC BID), or vehicle BID (2nd dose was administered approximately 6 hours after the 1st dose on each day) on day 1, that is, first dose around 24 hours post-infection, and day 2 post-inoculation. The mice were observed during the study and scored based on their behavior and clinical signs. Clinical scoring comprised assessment of piloerection in the skin, activity (movement, curiosity), and appearance of eyes. The scoring range comprised 0–4. In our model, a maximum score of 1; thus, only mild discomfort was observed. On day 3 post-inoculation and after sampling urine, mice were euthanized by cervical dislocation, and bladder and kidneys were removed from 21 mice in each treatment group aseptically for cfu determination. The bladder and kidneys were stored at −80°C and later homogenized in 0.5 or 1 mL saline, respectively. Colony counts of urine were determined immediately. From the three additional mice in each treatment group (satellite groups), urine and plasma were sampled and frozen at −80°C, and bladders, kidneys, and liver were collected in 4% buffered formalin. Cfu was determined in urine samples within 2–3 hours after sampling. Frozen organs were thawed and homogenized with steel beads using a tissue lyser. All samples, urine, kidney, and bladder, were 10-fold diluted in saline and 20 µL spots were applied on blue agar plates in duplicates. In addition, undiluted samples of urine (2–100 μL depending on the available amount of urine) were spread on a separate agar plate to determine the colony counts at the lowest possible detection level. All agar plates were incubated for 18–22 hours at 35°C in ambient air.

### Histopathological analysis

Approximately 3 µm thick sections of formalin-fixed, paraffin-embedded samples of liver and kidney were analyzed blinded, and randomized to the experimental groups by a trained veterinarian. Livers were analyzed for the parameters: glycogen (−1: slight reduction, −2 moderate reduction of 30%–70%, −3 no visible glycogen in more than 70% of cells), lymphocytic aggregates (0 = up to 10 small aggregates, 1 = 10–20 small aggregates, 2 => 20 small aggregates or up to two large aggregates, 3 = more than two large aggregates), anisocariosis (1 = mild anisocariosis, 2 = moderate anisocariosis, 3 = severe), double-nucleated cells (1 = sporadic, 2 = more than 3 per 40× field of view, 3 = more than 10 per 40× field of view). Kidneys were analyzed for changes in the glomeruli (not found), tubular dilation (1 = mild or up to 20% of tubuli affected, 2 = moderate or 25-40% of tubuli affected, 3 = severe 50% or more), and inflammatory infiltrate in the pelvis (1 = mild, 2 = moderate, 3 = severe).

### Statistical analysis

For the neutropenic thigh infection model with *E. coli* ATCC 25922, the thighs were regarded as two independent infection sites throughout the experiment. For statistical analysis, individual thighs (left and right) were treated as separate samples although these were not completely independent values. For the neutropenic thigh infection model with *K. pneumoniae* ATCC 43816, the individual thighs (left and right) were treated as one sample. For the efficacy models, the non-parametric Kruskal-Wallis test was used with GraphPad Prism 9.4.1. For the ascending urinary tract infection model, an ordinary one-way ANOVA with Dunnett’s multiple comparison test was used with GraphPad Prism 9.4.1.

## SUPPLEMENTAL MATERIAL

Supplemental material spectrum.01289-23-s0001.pdf Fig. S1 to S7 and Tables S1 to S7.

ASM does not own the copyrights to Supplemental Material that may be linked to, or accessed through, an article. The authors have granted ASM a non-exclusive, world-wide license to publish the Supplemental Material files. Please contact the corresponding author directly for reuse.
