# Supplementary material for: Pharmacokinetic and pharmacodynamic evaluation of the atypical tetracyclines chelocardin and amidochelocardin in murine infection models
Source: Microbiol Spectr. 2023 Dec 4;12(1):e01289-23. doi: 10.1128/spectrum.01289-23 (PMC10783034; doi:10.1128/spectrum.01289-23)
Supplement: Supplemental material — Fig. S1 to S7 and Tables S1 to S7. [file spectrum.01289-23-s0001.pdf]

Supplemental Information

Pharmacokinetic and pharmacodynamic evaluation of the atypical tetracyclines chelocardin and amidochelocardin in murine infection models

Running title: PK/PD profiling of chelocardin and amidochelocardin

Katharina Rox[1,2], Rolf Jansen[3], Tadeja Lukežič[2,4], Marina Greweling-Pils[5], Jennifer Herrmann[2,4], Marcus Miethke[2,4], Stephan Hüttel[2,3], Fabienne Hennessen[2,4], Antoine Abou Fayad[2,4], Cornelia Holzhausen[5], Carina Vingsbo Lundberg[6], Joanne Teague[7], Enge Sudarman[3], Lisa Bülter[2,8], Thomas Hesterkamp[2,8], Marc Stadler[2,3], Mark Brönstrup[1,2,#], Rolf Müller[2,4,#]

Table of content

Supplemental figures 3

Figure S1. LC-MS/MS traces of CHD, epi-CHD and CDCHD in plasma after IV administration. 3

Figure S2. Pharmacokinetic profiles of CHD and CDCHD at 15 and 30 mg/kg IV. 3

Figure S3. Assessment of low doses of CHD and CDCHD in the neutropenic thigh infection model with *K. pneumoniae* 4

Figure S4. Assessment of higher doses of CHD in the neutropenic thigh infection model with *K. pneumoniae*. 4

Figure S5. Assessment of higher doses of CDCHD in the neutropenic thigh infection model with *K. pneumoniae*. 5

Figure S6. Assessment of CHD and CDCHD in an ascending urinary tract infection model with display of the 95 % confidence interval. 5

Figure S7. Bioanalysis of terminal plasma and urine samples from the ascending urinary tract infection model. 6

Supplemental tables 7

Table S1. MIC determination of epi-CHD, CHD and CDCHD against a variety of ESKAPE pathogens. 7

Table S2. MIC of CHD, CDCHD, gentamicin, levofloxacin and ciprofloxacin against strains used for *in vivo* testing. 7

Table S3. Mass transitions for quantification of CDCHD and CHD using caffeine as internal standard. 7

Table S4. Doses and dosage regimens in the neutropenic thigh infection model with *K. pneumoniae* (low dose CHD/CDCHD). 7

Table S5. Doses and dosage regimens in the neutropenic thigh infection model with *K. pneumoniae* (high dose CHD). 8

Table S6. Doses and dosage regimens in the neutropenic thigh infection model with *K. pneumoniae* (high dose CDCHD). 8

Table S7. Doses and dosage regimens in the neutropenic thigh infection model with *E. coli*. 8

Supplemental figures

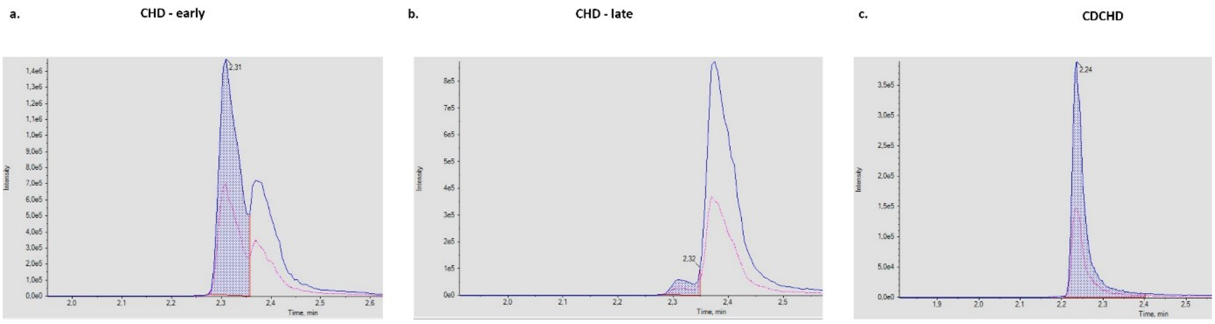

Figure S1. LC-MS/MS traces of CHD, epi-CHD and CDCHD in plasma after IV administration.

LC-MS/MS traces of CHD (a,b) and CDCHD (c) are displayed. CHD and epi-CHD in plasma are displayed 15 min (a) and 24 hours (b) post IV administration of 15 mg/kg. CDCHD in plasma is displayed 15 min after administration (c). The active epimer is marked in blue. Whereas the active epimer decreases for CHD (a,b), no epi-CDCHD can be found (c).

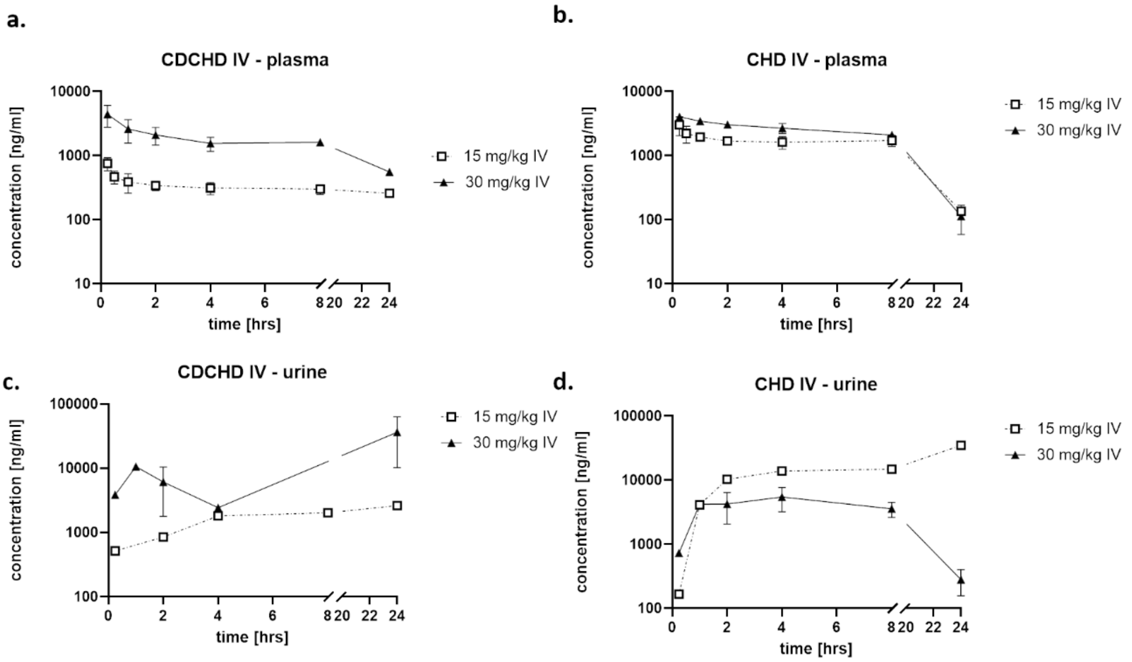

Figure S2. Pharmacokinetic profiles of CHD and CDCHD at 15 and 30 mg/kg IV.

CDCHD (a,c) and CHD (b,d) were administered at 15 mg/kg IV and 30 mg/kg IV, respectively. Plasma concentrations of CDCHD (a) and CHD (c) at 15 mg/kg IV (white) and 30 mg/kg IV (black) are displayed. Moreover, urine concentrations of CDCHD (b) and CHD (d) at 15 mg/kg IV (white) and 30 mg/kg IV (black) are displayed. N=3 mice were used per time point.

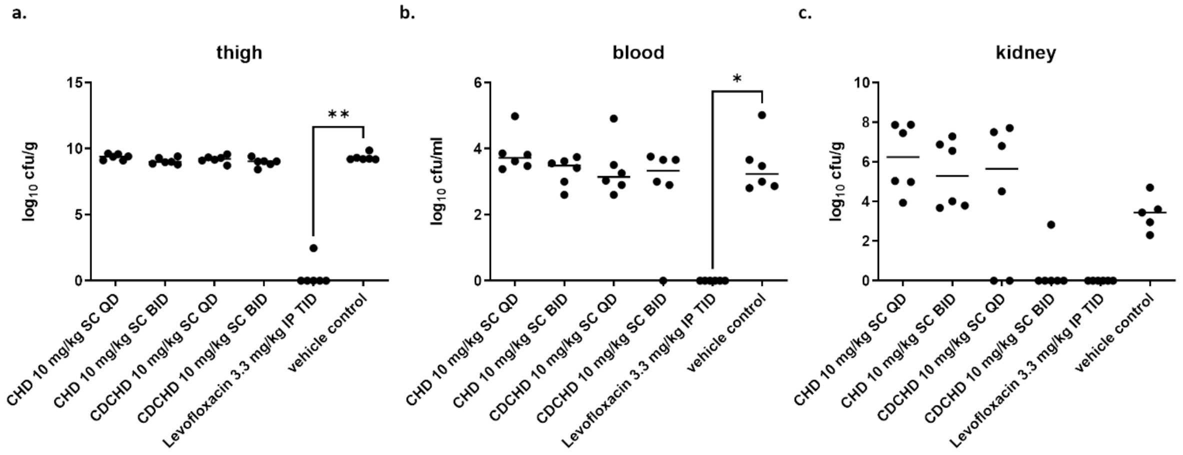

Figure S3. Assessment of low doses of CHD and CDCHD in the neutropenic thigh infection model with *K. pneumoniae*.

CHD and CDCHD were tested in the neutropenic thigh infection model with *K. pneumoniae* at 10 mg/kg SC QD and 10 mg/kg SC BID, respectively, against levofloxacin 3.3 mg/kg IP TID. Bacterial loads were determined in thigh (a), blood (b) and kidney (c). Bacterial loads were expressed as $\log_{10}$ cfu/g in thigh and kidney and as $\log_{10}$ cfu/ml in blood. Per group n=6 animals were used. Two thighs from one animal were pooled. *: $p < 0.05$, ** $p < 0.01$.

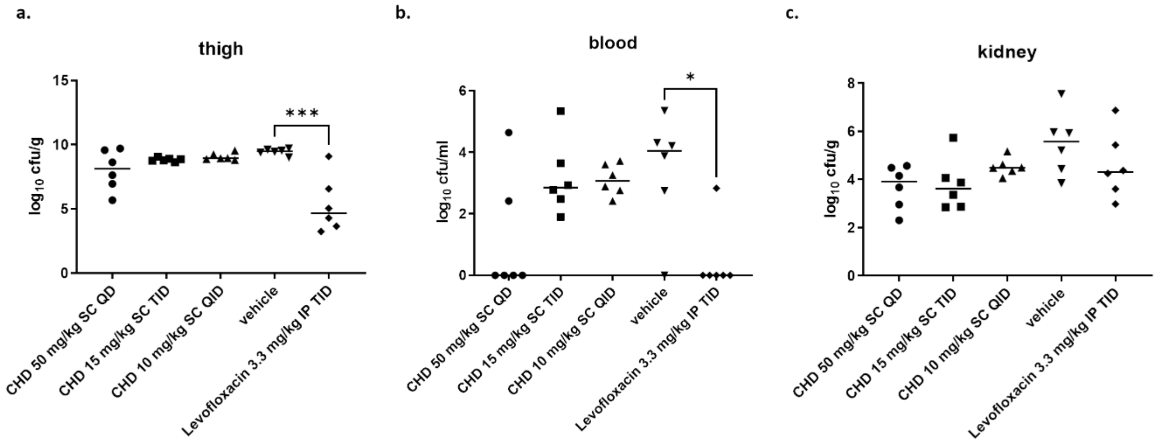

Figure S4. Assessment of higher doses of CHD in the neutropenic thigh infection model with *K. pneumoniae*.

CHD was tested in the neutropenic thigh infection model with *K. pneumoniae* at 50 mg/kg SC QD, 15 mg/kg SC TID and 10 mg/kg SC QID against levofloxacin 3.3 mg/kg IP TID. Bacterial loads were determined in thigh (a), blood (b) and kidney (c). Bacterial loads were expressed as $\log_{10}$ cfu/g in thigh and kidney and as $\log_{10}$ cfu/ml in blood. Per group n=6 animals were used. Two thighs from one animal were pooled. *: $p < 0.05$, ***: $p < 0.001$.

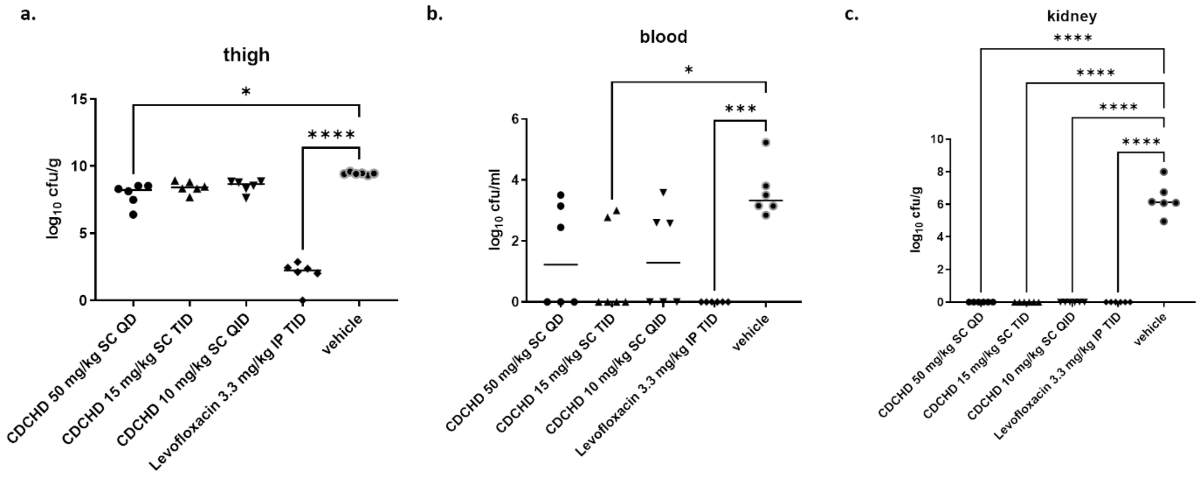

Figure S5. Assessment of higher doses of CDCHD in the neutropenic thigh infection model with *K. pneumoniae*.

CDCHD was tested in the neutropenic thigh infection model with *K. pneumoniae* at 50 mg/kg SC QD, 15 mg/kg SC TID and 10 mg/kg SC QID against levofloxacin 3.3 mg/kg IP TID. Bacterial loads were determined in thigh (a), blood (b) and kidney (c). Bacterial loads were expressed as $\log_{10}$ cfu/g in thigh and kidney and as $\log_{10}$ cfu/ml in blood. Per group n=6 animals were used. Two thighs from one animal were pooled. *: $p < 0.05$, ***: $p < 0.001$, ****: $p < 0.0001$.

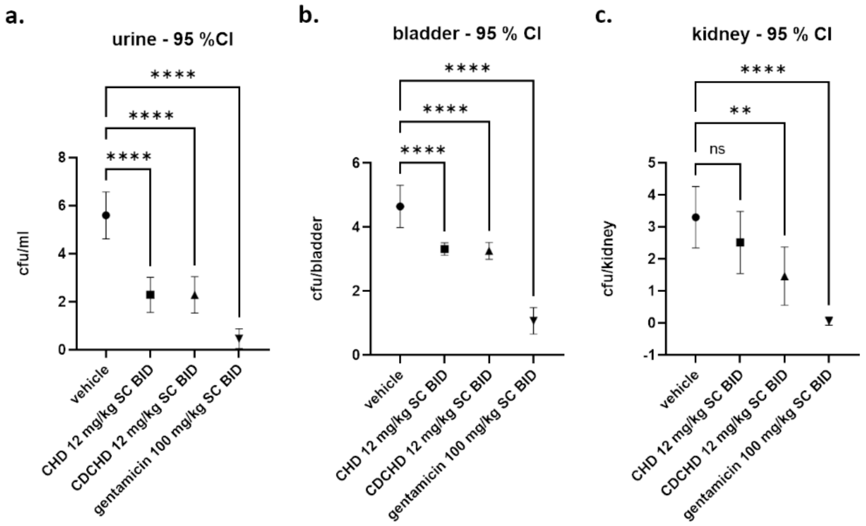

Figure S6. Assessment of CHD and CDCHD in an ascending urinary tract infection model with display of the 95 % confidence interval.

CHD and CDCHD were tested in an ascending urinary tract infection model with *E. coli* at 10 mg/kg SC BID against gentamicin 100 mg/kg SC BID. Bacterial loads were determined in urine (a), bladder (b) and kidney (c). Bacterial loads were expressed as $\log_{10}$ cfu/ml in urine, $\log_{10}$ cfu/bladder for bladder and as and kidney and as $\log_{10}$ cfu/kidney for kidney. Per group n=21 animals were used. Here, the mean with the 95% confidence interval (CI) is plotted. ns : not significant, **: $p < 0.01$, **** : $p < 0.0001$.

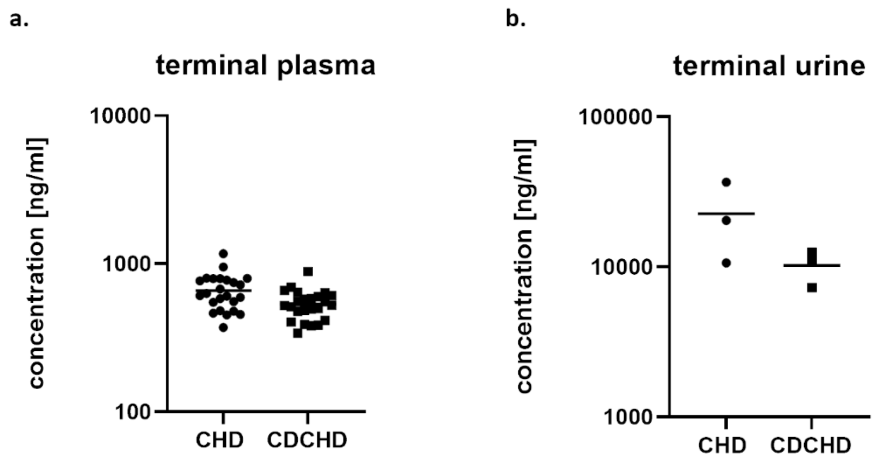

Figure S7. Bioanalysis of terminal plasma and urine samples from the ascending urinary tract infection model.

CDCHD and CHD were both assessed in an urinary tract infection model with *E. coli*. Terminal plasma (a) and urine (b) levels of both CHD and CDCHD are displayed. N=24 for plasma and n=3 for urine.

Supplemental tables

Table S1. MIC determination of epi-CHD, CHD and CDCHD against a variety of ESKAPE pathogens.

	MIC [µg/ml]		
**pathogen**	epi-CHD	CHD	CDCHD
***E. faecium* DSM-20477**	64	4	2
***S. aureus* DSM-346**	16	4	2
***K. pneumoniae* DSM-30104**	16	2-4	1
***A. baumannii* DSM-30008**	16	16	8
***P. aeruginosa* DSM-11128**	> 64	> 64	16
***E. aerogenes* DSM-30053**	64	32	8

Table S2. MIC of CHD, CDCHD, gentamicin, levofloxacin and ciprofloxacin against strains used for *in vivo* testing.

	MIC [µg/ml]				
**pathogen**	CHD	CDCHD	levofloxacin	ciprofloxacin	gentamicin
***K. pneumoniae* ATCC 43816**	5	1.25	0.08	-	-
***E. coli* ATCC 25922**	2	2	-	0.008	-
***E. coli* C175-94**	2	2-4	-	-	0.75

Table S3. Mass transitions for quantification of CDCHD and CHD using caffeine as internal standard.

	**Q1 mass**	**Q3 mass**	**DP [volts]**	**CE [volts]**	**CXP [volts]**
**Caffeine**	195.024	138.0	80.0	27.0	10.0
		110.0	80.0	31.0	6.0
**CDCHD**	413.069	396.2	80.0	21.0	26.0
		378.1	80.0	21.0	28.0
**CHD**	412.056	271.1	80.0	21.0	14.0
		253.1	80.0	33.0	16.0

DP: declustering potential; CE: collision energy; CXP: cell exit potential

Table S4. Doses and dosage regimens in the neutropenic thigh infection model with *K. pneumoniae* (low dose CHD/CDCHD).

**compound**	**dose [mg/kg]**	**route**	**time points [h]**
**vehicle**	-	SC	1, 3, 5, 7
**levofloxacin**	3.3	IP	2, 6, 10
**CHD**	10	SC	2, 10
**CHD**	10	SC	6
**CDCHD**	10	SC	2, 10
**CDCHD**	10	SC	6

Table S5. Doses and dosage regimens in the neutropenic thigh infection model with *K. pneumoniae* (high dose CHD).

compound	Dose [mg/kg]	Route	time points [h]
**vehicle**	-	SC	1, 3, 5, 7
**levofloxacin**	3.3	IP	2, 6, 10
**CHD**	10	SC	1, 3, 5, 7
**CHD**	15	SC	1, 4, 7
**CHD**	50	SC	1

Table S6. Doses and dosage regimens in the neutropenic thigh infection model with *K. pneumoniae* (high dose CDCHD).

compound	Dose [mg/kg]	Route	time points [h]
**Vehicle**	-	SC	1, 3, 5, 7
**levofloxacin**	3.3	IP	2, 6, 10
**CDCHD**	10	SC	1, 3, 5, 7
**CDCHD**	15	SC	1, 4, 7
**CDCHD**	50	SC	1

Table S7. Doses and dosage regimens in the neutropenic thigh infection model with *E. coli*.

compound	Dose [mg/kg]	Route	time points [h]
**Vehicle**	-	IV	1, 6
**ciprofloxacin**	20	IV	1, 9
**CHD**	15	IV	1, 6
**CDCHD**	15	IV	1, 6
**CHD**	50	IV	1
**CDCHD**	50	IV	1
